# Revisiting Secondary Information Related to Pharmacogenetic Testing

**DOI:** 10.3389/fgene.2021.741395

**Published:** 2021-10-01

**Authors:** Susanne B. Haga

**Affiliations:** Center for Applied Genomic and Precision Medicine, Duke University School of Medicine, Durham, NC, United States

**Keywords:** standards, patient consent, education, incidental (secondary) findings, clinical reporting

## Abstract

Incidental or secondary findings have been a major part of the discussion of genomic medicine research and clinical applications. For pharmacogenetic (PGx) testing, secondary findings arise due to the pleiotropic effects of pharmacogenes, often related to their endogenous functions. Unlike the guidelines that have been developed for whole exome or genome sequencing applications for management of secondary findings (though slightly different from PGx testing in that these refer to detection of variants in multiple genes, some with clinical significance and actionability), no corresponding guidelines have been developed for PGx clinical laboratories. Nonetheless, patient and provider education will remain key components of any PGx testing program to minimize adverse responses related to secondary findings.

## Introduction

Pharmacogenetic (PGx) testing has been implemented in a variety of clinical settings, including inpatient and outpatient settings (Cavallari et al., 2016; Schuh and Crosby, 2019; Smith et al., 2020; Dong et al., 2021), community pharmacies (Ferreri et al., 2014), academic medical centers (Hicks et al., 2012), executive health programs (Liko et al., 2021), and nursing homes (Dorfman et al., 2020). Despite the excitement for the field to improve therapeutic decision-making, the early adopters of PGx testing have highlighted some barriers, demonstrating the complexity of initiating a new type of testing with multiple types of delivery approaches, and limited provider awareness and clinical decision support (Klein et al., 2017; Moyer and Caraballo, 2017; Lanting et al., 2020; Omer, 2020; Chang et al., 2021; Luczak et al., 2021). The debate about the value of PGx testing continues as more evidence is gathered (Davis et al., 2021; Hicks et al., 2021), impacted by when and where testing is delivered, the type of test, and cost-effectiveness (Janssens and Deverka, 2014; Plumpton et al., 2019). In contrast to disease-based genetic testing, PGx tests are perceived to raise fewer ethical and psychosocial concerns (Peterson-Iyer, 2008; Gershon et al., 2014; Haga, 2009; Meli et al., 2021) than disease-based testing and do not typically require the involvement of genetic specialists or intensive patient education and counseling. However, one of the ethical concerns about PGx testing is the potential for additional information to be revealed, known as incidental or secondary findings, (Henrikson et al., 2008; Westbrook et al., 2013). Secondary findings may be welcome for some patients and undesired by others, but regardless of preference, their management warrants consideration by clinical testing laboratories and health providers to optimize patient and provider comprehension and respect for patient preferences.

### PGx Secondary Findings vs. Other Secondary Findings

Secondary findings are not unique to PGx testing and differ slightly from other types of clinical testing. For example, asymptomatic masses detected on imaging are referred to as “incidentalomas.” The frequency of incidentalomas varies by tissue (Vernooij et al., 2007; Secchi et al., 2017; Lu et al., 2021).

In pathology, and specifically with respect to whole genome sequencing (WGS) or exome genome sequencing (WES) (analogous to a whole body scan in radiology), secondary findings occur with the detection of genetic variants throughout the genome or exome that are unrelated to the clinical indication for testing (Amendola et al., 2015). Secondary findings have also been reported with other types of comprehensive genetic and genomic applications including non-invasive prenatal testing (Bianchi et al., 2015; Mastromoro et al., 2021) and chromosomal microarray testing (Rosina et al., 2021). Although much of the literature has focused on secondary findings in the germline, it is also possible to detect secondary findings present in somatic tissues in germline testing due to mixed cell populations in the patient specimen (Weitzel et al., 2018; Chao et al., 2021). Likewise, tumor-based testing can reveal secondary germline findings (Cushman-Vokoun et al., 2021). In some cases, disease-related genes are also associated with medication responses (Hosoya and Miyagawa, 2021).

In contrast, for PGx testing, secondary findings occur due to the pleiotropic effects of some pharmacogenes, where a gene associated with a given medication response for which testing is ordered is also linked to another phenotype, either response to other medications and/or disease risks, both unrelated to the current clinical indication (therefore, same gene but multiple phenotypes). ApoE is often cited as an example of a secondary finding for PGx testing, due to its association with both statin response and risk of Alzheimer’s disease (Bainbridge et al., 2011). Furthermore, PGx testing differs from other tests in that the scope of testing is limited to the analysis of one or more genes associated with a given medication response, and therefore, the potential for discovery of a genetic variant in a gene not related to drug metabolism or other function is not possible. Thus, due to the nature of the test, certain genes like ApoE can be excluded in a PGx test, a gene which is not typically included in PGx test panels (Haga and Kantor, 2018).

### Identification and Management of PGx Secondary Findings

These examples highlight the complexity and range of information revealed by secondary findings and the dilemmas presented with respect to the appropriate reporting, management and follow-up. Several clinical guidelines have been developed in radiology for the management of incidental findings for different tissues (Fassnacht et al., 2016; Patel et al., 2020).

With the increasing use of WGS/WES in the 2010s, several professional organizations have recognized the potential for secondary findings and/or developed guidance on how to manage these variants (van El et al., 2013; Boycott et al., 2015; Matthijs et al., 2016). In 2013, the American College of Medical Genetics and Genomics (ACMG) issued its first guideline regarding the reporting of secondary findings detected in WES/WGS testing (Green et al., 2013). A total of 56 genes were selected for which the evidence linked to a given phenotype is strong and the ability to intervene exists (i.e., “actionable”). Clinical testing laboratories can include an addendum to the WES/WGS test report of the additional genes if patients consent to receive the secondary report. The list has been revised twice: in 2017, the list was updated to include 59 genes (Kalia et al., 2017) and in 2021, 14 more genes were added to bring the total to 73 genes (Miller et al., 2021). Several papers have reported the identification of pathogenic/actionable PGx variants from WES/WGS datasets (Lee et al., 2016; Thauvin-Robinet et al., 2019; Eghbali et al., 2020). The 2017 ACMG guidelines indicated that PGx variants were being considered for inclusion in the future but none were considered for the 2021 guidance. Both RYR1 and CACNA1S are already included on the ACMG list (for malignant hyperthermia susceptibility) for which a pharmacogenetic guideline has been developed (Gonsalves et al., 2019). In patients undergoing WES/WGS, the detection of variants from the ACMG gene list in non-high-risk patients and PGx variants has raised the possibility of population health screening for some of these genes (Levy-Lahad et al., 2015; Rego et al., 2018; Chaudhari et al., 2020), though they have not been validated for this purpose (ACMG Board of Directors, 2019).

Since PGx testing is limited to a relatively small set of genes associated with metabolism, transport and other pathways important to medication response (Haga and Kantor, 2018), the potential for (and quantity of) secondary information is obviously less than WGS/WES or other broad-based testing platforms, though not insignificant. In a 2013 publication, Westbrook at el. conducted an extensive literature review to define the extent of PGx incidental findings. Based on a 34-gene PGx test panel, they identified 26 genes with a reported secondary finding and eight of these genes had secondary findings replicated (Westbrook et al., 2013). Each gene had an average of 11 reported associated phenotypes of statistical significance, but only 0.4 associated phenotypes were validated. For example, ABC1 has been associated with breast cancer, colorectal cancer, and inflammatory bowel disease. Furthermore, they reported extensive variability in the number of studies with respect to racial and ethnic diversity, with substantially fewer replicated studies for non-European groups. In 2016, Oetjens et al. analyzed 184 functional variants in 34 pharmacogenes and reported five replicated genotype-phenotype associations and identified an additional eight novel associations (Oetjens et al., 2016).

The Clinical Pharmacogenetics Implementation Consortium (CPIC) has developed 25 guidelines on the interpretation and recommendations for use of PGx test results. In each guideline, there is a section entitled “Incidental Findings” for either the gene and/or drug (or both). A review of the 25 current CPIC guidelines finds eight guidelines that mention a disease risk or phenotype (not counting CFTR for the medication ivacaftor since it is a diagnostic test for cystic fibrosis) (Table 1). Of the six guidelines on or that include CYP2D6 (Hicks et al., 2015; Bell et al., 2017; Hicks et al., 2017; Goetz et al., 2018; Brown et al., 2019; Crews et al., 2021) aside from implications for other therapies, only one noted incidental findings related to a disease risk (suicide and depression).

**TABLE 1:** Incidental findings in CPIC guidelines.

Gene (medication)	Incidental finding
CYP2D6 (SSRIs)	Suicide/Depression
G6PD (rasburicase)	Adverse response to fava beans, neonatal hyperbilirubinemia, Gilbert’s syndrome
IFNL3 (peg interferon)	Variant rs12979860 linked to HCV-induced hepatocellular carcinoma and graft fibrosis, allergic disease in children, liver fibrosis, viral cirrhosis due to HCV, and greater likelihood of HCV persistence, particularly in HCV genotypes 1 and 4. The rs12979860 CC genotype is associated with lower frequency of hepatic steatosis in patients with chronic HCV; carriers of T allele linked to increased susceptibility to chronic hepatitis B virus infection and hepatocellular carcinoma compared with non-carriers
UGT1A1 (atazanavir)	Gilbert syndrome; Crigler-Najjar syndrome type 1; Crigler-Najjar syndrome type 2
VKORC1 (warfarin)	Homozygosity for rare coding mutations in VKORC1 cause combined deficiency of vitamin K dependent clotting factors-2 (VKCFD2)
DPYD (fluoropyrimidines)	Patients homozygous for inactivating variants of DPYD have complete dihydropyrimidine dehydrogenase deficiency
HLA-B*57:01 (abacavir)	HIV viral load; HIV long-term non-progressors
RYR1 (sevoflurane, halothane, enflurane, isoflurane, methoxyflurane, and desflurane, succinylcholine)	Myopathies; central core disease, multiminicore disease, congenital fiber type disproportion, centronuclear myopathy, King-Denborough syndrome, nemaline myopathy, and congenital myopathy with cores and rods

### Patient Perspectives

As clinical evidence continues to accumulate, the number of associations of PGx variants (and non-PGx variants) with responses to multiple medications as well as disease risk linked to their endogenous roles (Nebert and Dalton, 2006) will likely expand as presaged by the growing ACMG list. Although secondary findings have been known for some time, reports of different group’s experiences with implementing PGx testing do not mention challenges related to management of secondary findings (Pasternak et al., 2020; Luczak et al., 2021). This lack of mention may be due to inadequate evidence of many PGx secondary findings, the limited number of validated secondary findings, or a lack of reporting by clinical PGx testing laboratories (due to absence of standards on reporting).

The discovery or reporting of secondary findings may cause some patient anxiety, confusion, additional expenses, and burden on the healthcare system, if it follows the experiences of some other genetic and genomic secondary findings. Compared to a clinical sequencing test for diagnosis, PGx testing may be viewed more as an elective test and the benefits and risks of the primary and secondary findings will be weighed differently. Thus, understanding the impact of secondary findings on PGx test utilization and patient interest in secondary findings will help inform implementation strategies. A number of studies have explored patient’s (Gray et al., 2016; Mackley et al., 2017; Hicks et al., 2018; Delanne et al., 2019; Houdayer et al., 2019) and research participants’ (Bollinger et al., 2012) interest in secondary findings related to clinical sequencing, reporting mixed interest in secondary findings, though generally favorable for those that are considered to be clinically actionable. While attitudes about PGx testing overall are generally favorable, much less exploration of secondary findings has been conducted (Haga et al., 2011).

## Discussion

So, where are we left with respect to secondary findings and PGx testing as it stands now? Medication response is a complex phenotype impacted by both host genetics, environmental factors and the gut microbiome. A growing body of literature highlights the important role of the gut microbiome on drug response (Zhang et al., 2018; Clarke et al., 2019) as well as disease risk (Andrews et al., 2021; Zhang et al., 2021). Thus, we should not confine the definition of secondary findings to host genetic variants. Future PGx testing that includes testing of both the host genome and the gut microbiome will likely produce more secondary findings. Further adding to the complexity of data interpretation are the interactions with diet and co-medications, leading to investigations into the potential use of artificial intelligence-based prediction tools (Lin et al., 2020; de Jong et al., 2021).

To date, no standards for the reporting of secondary findings for clinical PGx testing have been developed. The path taken by the clinical sequencing community and experiences to date can inform the development of a similar approach by the PGx community. For clinical sequencing, the ACMG guideline recommends offering secondary findings to all patients irrespective of age as an opt-out (Miller et al., 2021).

A first step would be to develop a consensus list of secondary findings of pharmacogenes to avoid disparities in what information is offered or reported between laboratories (Bombard et al., 2020; Reble et al., 2021). Then, clinical PGx testing labs that include genes in their test panels with secondary findings could offer patients the option of receiving a separate report on secondary findings (Brothers et al., 2017).

However, the clinical setting in which PGx testing is offered is likely to be quite different from that in which clinical sequencing is offered with respect to the types of providers, their knowledge of genetics, and time to discuss testing with patients. Specifically, genetic specialists are not typically involved in the delivery of PGx testing and thus, the delivery of information for PGx testing (and primary and secondary test results) will be quite different. Development of patient educational materials are essential, including information about secondary findings in the informed consent forms, though at the time of our review of informed consent documents, we did not find any mention of secondary findings (Haga and Mills, 2016). Efforts should be directed toward novel patient communication strategies such as through videos or step-by-step navigational tools to explain testing or how to understand the lab report for both primary and secondary findings (if requested).

Education of providers will remain a critical component to promoting informed decision-making about testing and preferences for secondary findings. Educational support is needed for not only authorized prescribers, but other health professionals such as nurses and pharmacists. While clinical decision supports provide an important component in the appropriate use of PGx testing (Nishimura et al., 2015; Liu et al., 2021), they may not be widely accessible or as helpful with respect to secondary findings. In some cases, the PGx test-ordering provider may not be the appropriate person to communicate secondary findings and referral to a pharmacist or a genetic counselor for secondary information related to other medications or disease risk, respectively, may be warranted (Callard et al., 2012; Zierhut et al., 2017; Chart et al., 2021). Patients may be directed to access their secondary results through their primary care provider, and with the patient’s consent, copies of the test results may be sent to both the test-ordering provider and a primary care provider. Alternatively, team-based genetics groups may be established to provide immediate support for a range of secondary findings revealed by all types of genetic and genomic testing including PGx testing to both patients and their providers (Thauvin-Robinet et al., 2019).

Furthermore, with the reported benefit of re-analysis and re-interpretation of variant data to reflect new evidence (Connell et al., 2019; Liu et al., 2019; Salfati et al., 2019; Neubauer et al., 2021), updated test reports may include new information regarding primary or secondary findings. Thus, in addition to obtaining patient preferences regarding current secondary findings, laboratories may consider soliciting patient preferences about receiving updated reports for both primary and secondary findings. In time, this may become standard practice for genetic and genomic testing.

In conclusion, the identification of disease-related secondary information of PGx variants presents a more complicated scenario for PGx testing, albeit still potentially beneficial.

Whether it is an incidental finding or not has almost become incidental in the debate about the management of these findings, eclipsed by whether the results are clinically actionable or show clinical utility. Though portrayed more as a risk of testing (or any other clinical tests), secondary findings may or may not be perceived as such by patients and informing patients in advance of the possibility is the best strategy. PGx testing represents a microcosm of the larger issue of secondary findings for WGS/WES and other comprehensive genomic analysis (e.g., NIPT). Though the scale differs, the major concerns overlap, notably how best to manage secondary results and standards for identification and reporting. In time, providers and patients may begin to view genetic and genomic testing as they might with radiology and the unavoidable detection of variants outside of the clinical indication—a “side effect” of PGx testing. But until that time, provider and patient preparation are key to minimizing adverse responses to secondary findings.

## Data Availability

The original contributions presented in the study are included in the article, further inquiries can be directed to the corresponding author.
